# Plant subtilases as key initiators of peptide ligand‐receptor signaling

**DOI:** 10.1111/tpj.70875

**Published:** 2026-04-15

**Authors:** Sayaka Matsui, Yuki Hirakawa

**Affiliations:** ^1^ Graduate School of Integrated Sciences for Life Hiroshima University Higashi Hiroshima Hiroshima 739‐8526 Japan

**Keywords:** protease, *Arabidopsis thaliana*, peptide ligand, proteolytic processing, substrate specificity

## Abstract

Peptide ligand‐receptor signaling plays a crucial role in regulating diverse biological processes in plants. Over the past three decades, numerous peptide ligands and their corresponding receptors have been identified, and our understanding of their downstream signaling pathways and biological functions has gradually advanced. By contrast, relatively less attention has been paid to an earlier step in the signaling process: the generation of active peptide ligands. Some peptide ligands are generated through proteolytic processing of inactive precursor proteins. In recent years, significant progress has been made in identifying the proteases involved in this maturation process in flowering plants, primarily *Arabidopsis thaliana*. In this review, we focus on the processing proteases of peptide ligands, particularly subtilases, identified to date. We discuss recent findings on these proteases, including the approaches used for their identification, their enzymatic activities and substrate specificities, and the regulation of protease activity involved in peptide maturation. These insights deepen our understanding of how peptide ligand‐receptor signaling pathways are initiated and ultimately provide important clues to the diverse physiological processes mediated by these signaling pathways.

## Significance Statement

The peptide ligand–receptor interactions depend on the specific peptide structures; however, relatively little has been known about peptide ligand formation, specifically the proteolytic processing of precursor proteins. This review summarizes the recent identification of several proteases, particularly subtilases, that are involved in peptide ligand processing, using both conventional and novel techniques. These findings contribute to our understanding of the molecular features of peptide ligand processing and its regulation, mediated by intrinsic and extrinsic cues, in the initiation of peptide signaling.

## INTRODUCTION

Peptide ligands and their corresponding receptors are key components of cell‐to‐cell communication. They are involved in a wide range of biological processes, including growth, development, reproduction, interactions with other organisms, and responses to environmental stimuli. Since the identification of systemin in tomato in 1991 (Pearce et al., 1991), numerous peptide‐receptor pairs have been identified, revealing their functional diversity across various biological contexts (Tavormina et al., 2015). Furthermore, genes encoding peptide ligands and their receptors have been identified in a variety of land plants, indicating that peptide‐mediated signaling is widely conserved across land plants (Furumizu & Shinohara, 2024).

**Figure 1:**
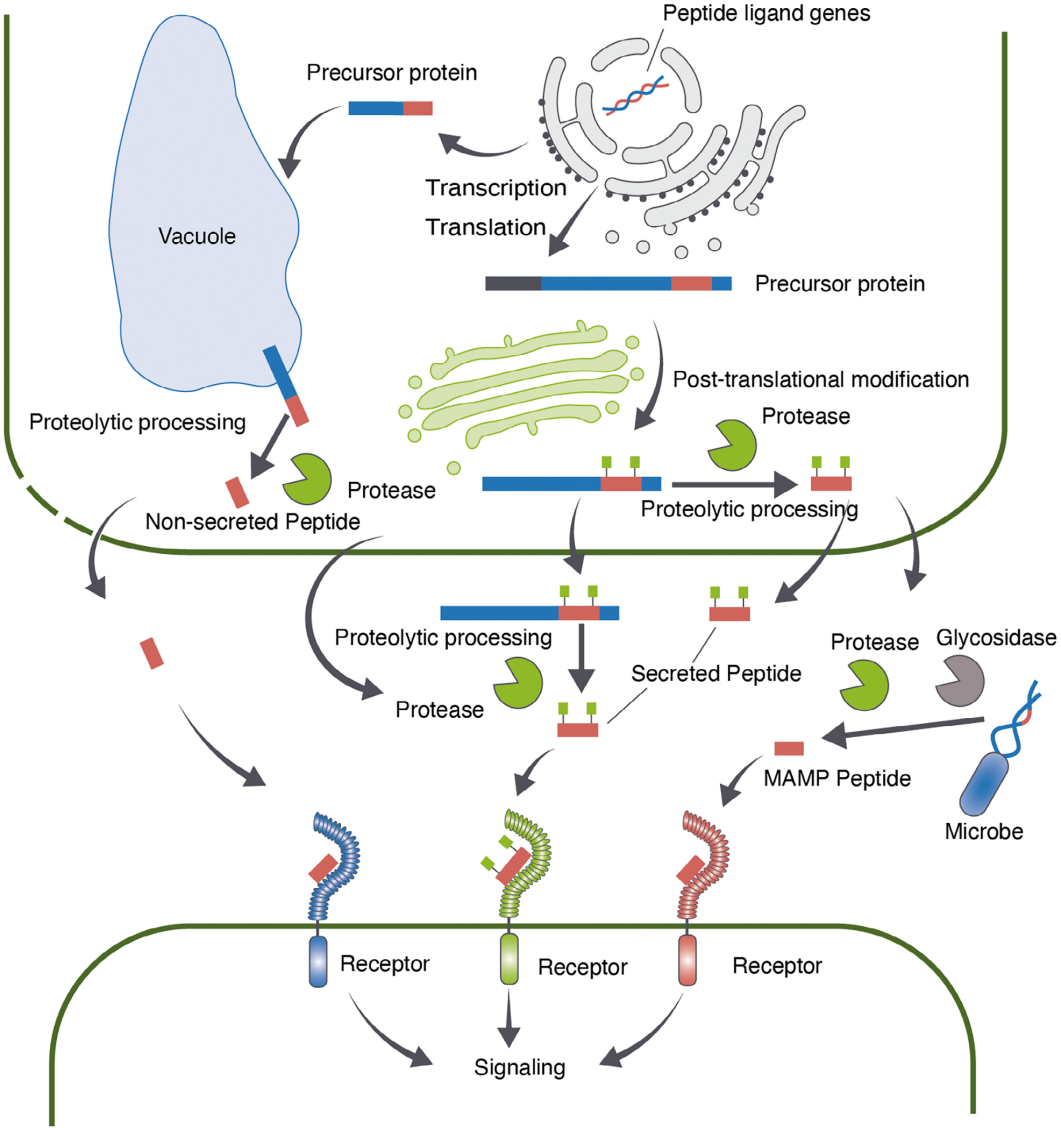
Primary pathways for peptide ligand generation. Endogenous peptide ligands are classified into secreted and non‐secreted types based on their production pathways. The production process of secreted peptides is shown in the center. The precursor protein is represented by a thick bar, with the signal peptide shown in gray, the propeptide in blue, and the mature peptide in red. The green squares indicate post‐translational modifications. The production of the non‐secreted peptide, PROPEP1, is shown on the left. This peptide is released outside the cell through damaged regions of the plasma membrane, indicated by dashed lines, and functions as a signaling molecule. The exogenous peptide ligand MAMP is shown on the right, illustrating the case of flg22 derived from flagellin. In the extracellular space, microbial molecules are fragmented through the action of proteases and other hydrolytic enzymes such as glycosidases, releasing peptide ligands.

**Figure 2:**
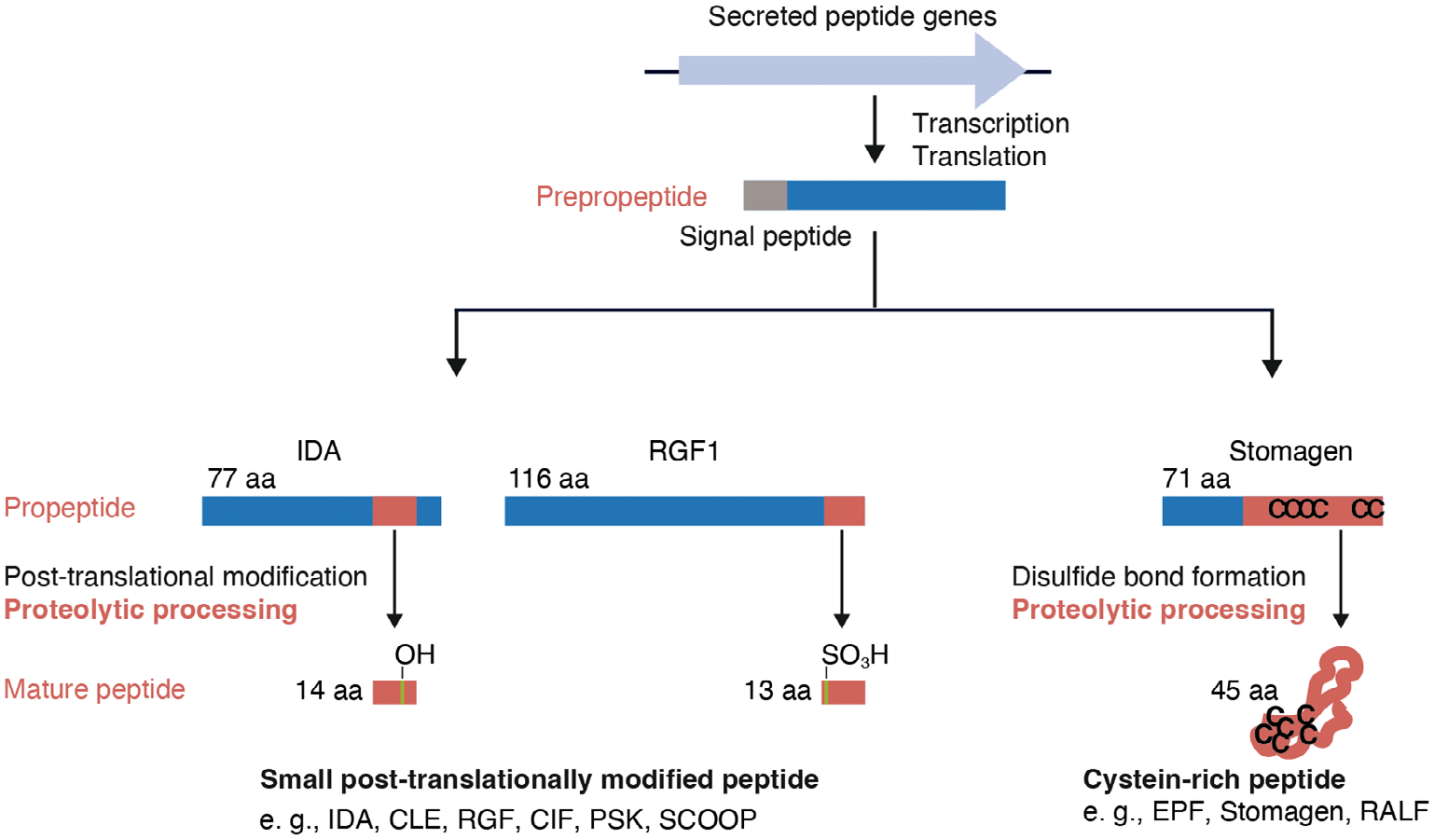
Classification of secretory peptides based on the structure. Secretory peptides are transcribed and translated as inactive precursors. Small post‐translationally modified peptides, which undergo post‐translational modification and proteolytic processing to mature, are shown on the left. IDA and RGF1 are shown as examples. Cysteine‐rich peptides, which form disulfide bonds within the molecule and undergo proteolytic processing to mature, are shown on the right. Stomagen is shown as an example (Matsubayashi, 2011).

Some peptide ligands act as peptide hormones, which are produced in plants and recognized by plant cell surface receptors. Peptide hormones are classified into secreted and non‐secreted peptides. Secreted peptides have an N‐terminal signal peptide sequence that directs the peptide to the extracellular space through the conventional secretory pathway, whereas non‐secreted peptides can also be released into the extracellular space through alternative mechanisms and act as signaling molecules (Figure 1). Secreted peptide hormones are further divided into two major groups: small post‐translationally modified peptides and cysteine‐rich peptides (Figure 2) (Matsubayashi, 2011). Small post‐translationally modified peptides are generally less than 20 amino acid residues in length and contain post‐translational modifications such as tyrosine sulfation, proline hydroxylation, and hydroxyproline arabinosylation. Cysteine‐rich peptides contain cysteine residues that form intramolecular disulfide bonds. Both groups of secreted peptide hormones are initially translated as biologically inactive prepropeptides, followed by the removal of the N‐terminal signal peptide to produce propeptides. The propeptides then undergo structural modifications and proteolytic processing by several enzymes to generate the mature peptides with specific lengths (Figure 2) (Matsubayashi, 2011).

Another type of peptide ligand is a microbe‐derived peptide, referred to as pathogen/microbe‐associated molecular patterns (PAMPs/MAMPs) (Figure 1). MAMPs are conserved molecular patterns specific to microbes and are recognized by plant pattern recognition receptors (PRRs), triggering plant immune responses (Boller & Felix, 2009). Peptide MAMPs are parts of microbial proteins that are critical for microbial survival or pathogenicity. Because MAMPs are often buried within these proteins, proteolytic release of the peptides from their precursors is thought to be required for their recognition by PRRs (Chen et al., 2024).

In this way, many peptide ligands are generated through proteolytic processing of inactive precursor proteins. Although our understanding of peptide ligand generation has been limited compared to that of ligand‐receptor interactions and downstream signaling, proteases involved in peptide ligand processing have been increasingly identified over recent years, the majority of which are classified into subtilisin‐like serine proteases (subtilases, SBTs) (Stintzi & Schaller, 2022; Stührwohldt & Schaller, 2019). This review focuses on subtilases responsible for processing peptide ligands. In the following sections, we discuss subtilases identified as being involved in the processing of specific peptide ligands, including the structural and biological features of the peptide ligands, the rationale and evidence for protease identification, and the molecular characterization of proteases, such as their cleavage sites, substrate specificity, and regulation of activity. Based on these examples, we discuss the functional features of subtilases as key initiators of peptide ligand‐receptor signaling. These insights contribute to a better understanding of the initiation of peptide ligand‐receptor signaling and the biological processes mediated by this signaling in plants (Boxes 1 and 2).

Box 1 **Bullet‐point summary**

- In recent years, an increasing number of proteases that cleave peptide ligands from their precursors have been identified in plants.
- The substrate specificity of proteases involved in peptide ligand processing ranges from highly stringent to relatively flexible.
- The activity of proteases involved in peptide ligand maturation is regulated by various physicochemical factors.
- Novel activity‐based protease identification methods have identified peptide ligand‐processing proteases, but complementary approaches are still required to discover additional ones.

Box 2 **Open questions**

- What are the molecular identities of the proteases involved in peptide ligand maturation that remain unidentified, and what strategies can be used to reveal them?
- How are processing proteases regulated in response to developmental cues, environmental stimuli, and other physiological signals to participate in peptide ligand signaling?
- Considering the complexity of the *in vivo* environment, where a single protease can cleave multiple substrates, what are the actual abundance, spatial distribution, and substrate interactions of the protease, and how might it coordinate and regulate multiple peptide signaling pathways to contribute to the overall biology of the plant?

## IDA

Inflorescence Deficient in Abscission (IDA) is a small post‐translationally modified peptide involved in the floral organ abscission process (Butenko et al., 2003). The *IDA* gene encodes a 77‐amino acid protein with a signal peptide, and the mature IDA peptide is proteolytically released (Butenko et al., 2003; Stenvik et al., 2008). The IDA peptide binds to the extracellular leucine‐rich repeat (LRR) domain of the receptor kinases (RKs) HAESA (HAE) and HAESA‐LIKE2 (HSL2), and promotes the association of these receptors with their co‐receptors, which belongs to the SOMATIC EMBRYOGENESIS RECEPTOR KINASE (SERK) family. This activates the intracellular MAPK cascade, which triggers the floral organ abscission process (Cho et al., 2008; Jinn et al., 2000; Meng et al., 2016; Santiago et al., 2016; Stenvik et al., 2008).

A loss‐of‐function approach involving the tissue‐specific expression of the subtilase inhibitors EPI1 and EPI10 from the oomycete *Phytophthora infestans* revealed that SBT activity is required for the floral organ abscission, and that SBT4.12, SBT4.13, and SBT5.2 cleave the IDA precursor to release the 14‐amino acid mature IDA peptide (Schardon et al., 2016). Peptide cleavage assays using SBT4.13 revealed that SBT4.13 likely recognizes an extended sequence motif including residues on both sides of the scissile bond, rather than a single amino acid at the P1 position (immediately upstream of the scissile bond). For the N‐terminal cleavage site of the mature IDA (YLPK‐GVPIPP; − represents the scissile bond), proline at the P2 position (Pro^54^) and tyrosine at the P4 position (Tyr^52^) were confirmed as the two most important residues for substrate recognition (Schardon et al., 2016).

## CLE

CLAVATA3 (CLV3) is a founding member of the CLE (CLV3/ESR‐related) family of small post‐translationally modified peptides (Fletcher et al., 1999). CLV3 peptides are perceived by the CLV1/BARELY ANY MERISTEM (BAM) family of LRR‐RKs, and their co‐receptors CLAVATA3 INSENSITIVE RECEPTOR KINASES (CIKs) to control the expression of WUSCHEL (WUS) and regulate the homeostasis of stem cell population during the shoot meristems in vegetative and reproductive growth phases (Brand et al., 2000; Hu et al., 2018; Nimchuk, 2017; Schoof et al., 2000; Shinohara et al., 2012). The CLV3‐WUS pathway is associated with quantitative trait loci (QTLs) for yield traits in crop plants such as maize, rice, and tomato (Somssich et al., 2016). The mature CLV3 peptide has been identified as a 13‐amino acid arabinosylated glycopeptide by the proteomic analysis of apoplastic peptides from *A. thaliana* plants overexpressing the *CLV3* gene (Ohyama et al., 2009). The CLV3 peptide is located at the CLE domain near the C‐terminus of the CLV3 protein. The proteolytic processing activity of CLV3 has been detected in plant extracts or cultured medium of *A. thaliana* and other species. While the trimming of the C‐terminus of the CLV3 peptide is suggested to occur by the action of progressive carboxypeptidase, its N‐terminus may be processed by specific endopeptidases since the 5‐amino acid long N‐terminal flanking sequence is critical for the efficient cleavage (Ni et al., 2011; Xu et al., 2013). However, although the activity for N‐terminal processing of CLV3 was detected, the proteases that cleave the proprotein at the N‐terminus of the CLV3 peptide have not been identified yet.

In *A. thaliana*, there are more than 30 genes encoding CLE precursors, some of which contain C‐terminal extensions in the preproproteins. Mature CLE peptides are 12‐ or 13‐amino acid peptides with proline hydroxylation and additional arabinosylation (Ito et al., 2006; Ohyama et al., 2009). SOL1 (SUPPRESSOR OF LLP1 1) is a single copy Zn^2+^‐carboxypeptidase identified in a genetic screening for suppressors of the short‐root phenotype of *CLE19* overexpression (Casamitjana‐Martínez et al., 2003). SOL1 has an *in vitro* carboxypeptidase activity against the C‐terminal arginine and lysine (Tamaki et al., 2013). The C‐terminal arginine or lysine residue immediately following the CLE peptide sequence is shared in several CLE proteins, which may also be processed by SOL1 *in vivo*. Endosomal localization of SOL1 proteins suggests that the C‐terminal processing occurs in the secretory pathway.

CLE40 is the closest homolog of CLV3 in the *A. thaliana* CLE peptide family and regulates stem cell fate in the root and shoot meristems (Berckmans et al., 2020; Schlegel et al., 2021). An inhibitor‐based loss‐of‐function approach revealed that the subtilases SBT1.4, SBT1.7, and SBT4.13 are required for the formation of the mature CLE40 peptide (Stührwohldt, Ehinger, et al., 2020). Interestingly, the SBTs also cleave CLE40 internally (RQVPT‐G‐SDPLHH), thereby inactivating the peptide. This activity is inhibited by the hydroxylation at Pro^65^ in vicinity of the scissile bond, a modification found in the mature CLE40 peptide, showing that the post‐translational modification can protect the precursor from SBT‐mediated cleavage at the internal processing site.

## RGF/GLV/CLEL

The ROOT GROWTH FACTOR (RGF)/GOLVEN (GLV)/CLE‐Like (CLEL) family of small post‐translationally modified peptides has been shown to be involved in root development in *A. thaliana* (Matsuzaki et al., 2010; Meng et al., 2012; Whitford et al., 2012). RGF was identified as a 13‐amino acid–sulfated peptide that complements the defects in root stem cell maintenance in the tyrosylprotein sulfotransferase mutant *tpst‐1* (Matsuzaki et al., 2010) and is perceived by the RGFR/RGI receptors (Ou et al., 2016; Shinohara et al., 2016; Song et al., 2016). A member of the RGF/GLV/CLEL family, CLEL6, is involved in gravitropism together with the functionally redundant CLEL9 peptide. A suppressor screen has identified SBT6.1 as a protease essential for CLEL6 processing, although the SBT6.1 cleavage site is located away from the N‐terminus of mature peptide motif (Ghorbani et al., 2016). Stührwohldt, Scholl, et al. (2020) showed the involvement of subtilases in the maturation of the CLEL6 and CLEL9 peptides using the inhibitor‐based loss‐of‐function approach driven by the promoters of CLEL6 and CLEL9. They further examined subtilases that are upregulated in the *tpst* mutant since the subtilases essential for the biogenesis of CLEL peptides may be upregulated as a compensatory response in the mutant lacking tyrosine sulfation essential for the function of the peptides. Among them, SBT3.8 has been identified to cleave the N‐terminal extension of CLEL6 peptide depending on the N‐terminal aspartate of the mature peptides. N‐terminally extended CLEL6 peptide was processed by SBT3.8 to generate mature CLEL6 at pH 5.5, but its activity was lost at pH 7.0, suggesting that processing occurs in acidic environments such as apoplast and post‐Golgi compartments.

## CIF

Casparian strip integrity factors (CIFs) are small post‐translationally modified peptides containing one sulfated tyrosine and two hydroxyproline residues. CIF1 and CIF2 are perceived by the LRR‐RK GASSHO1 (GSO1)/SCHENGEN3 and its homolog GSO2, and regulate the formation of the Casparian strip, a hydrophobic barrier on root endodermal cells (Doblas et al., 2017; Nakayama et al., 2017; Okuda et al., 2020). The CIF1 and CIF2 genes encode ~80‐amino acid polypeptides, and the 21‐amino acid mature peptides are derived from a conserved domain at the C‐terminus.

CIF3 and CIF4 are also derived from the conserved C‐terminal domain of ~80‐ to 100‐amino acid precursors. CIF3/4‐GSO1/2 signaling is required for pollen wall formation. Unlike CIF1 and CIF2, CIF3 and CIF4 possess C‐terminal extensions, suggesting that C‐terminal processing is required. Based on the strong and specific pollen‐localized expression from the onset of pollen wall formation through to pollen maturity, SBT5.4 has been identified as a CIF‐processing protease. Recombinant SBT5.4 cleaves the CIF4 precursor *in vitro*. A cleavage assay using a synthetic peptide extended by three precursor‐derived amino acid residues at both the N‐ and C‐termini of CIF4 revealed that SBT5.4 cleaves CIF4 at its C‐terminus, specifically between His^89^ and Gly^90^ (LYGDYGFWNPSPVYGGGFPYPGPVPH‐GSL). The following model has been proposed. SBT5.4 (and maybe other SBTs), expressed in pollen, processes CIF3/4 precursors produced in the tapetum. The mature CIF3/4 peptides diffuse between tapetum cells and binds to GSO receptors in the middle layer, thereby activating downstream signaling. This signaling induces pollen wall formation by organizing the secretion of pollen wall components. Completion of the pollen wall prevents further interaction between SBT5.4 and CIF precursors, thereby attenuating the signaling (Truskina et al., 2022).

*TWISTED SEED1* (*TWS1*) encodes an 81‐amino acid protein containing an N‐terminal signal peptide and a domain with limited similarity to CIF peptides (Doll et al., 2020; Fiume et al., 2016). TWS1 precursor also contains a C‐terminal extension. Similarities in seed‐twisting and cuticle‐permeability phenotypes in mutants prompted an investigation of the involvement of ABNORMAL LEAF SHAPE1 (ALE1; SBT2.4) in the processing of TWS1 (Creff et al., 2019; Fiume et al., 2016; Tanaka et al., 2001). It was revealed that ALE1 cleaves the TWS1 precursor both *in planta* and *in vitro*. The cleavage of the TWS1 precursor occurs at His^54^‐Gly^55^, where His^54^ corresponds to the C‐termini of CIF peptides. Substitution of either His^54^ or Gly^55^ abolished cleavage, indicating that these amino acids are important for ALE1‐mediated processing. A model has been proposed for monitoring embryonic cuticle integrity. Before the completion of gap‐filling, the TWS1 precursor is secreted from the embryo into the apoplast, where it undergoes processing by ALE1 (and likely other subtilases) secreted from the endosperm cells. The mature TWS1 peptide binds to GSO receptors on the embryo surface and promotes cuticle deposition. When the cuticle is intact, it spatially separates ALE1 from the TWS1 precursor, thereby attenuating the signal (Doll et al., 2020). To identify the protease responsible for N‐terminal cleavage of the TWS1 peptide, Royek *et al*. searched for proteases coexpressed with ALE1 in developing *A*. *thaliana* seeds and identified SBT1.8 as a candidate. They showed that SBT1.8 cleaves the C‐terminus of the TWS1 peptide redundantly with ALE1 and additionally cleaves the N‐terminus of the TWS1 peptide at Glu^31^‐Asp^32^ (MKVGLE‐DYNFPV). Interestingly, the specificity of N‐terminal cleavage by SBT1.8 required tyrosine sulfation at the P2' position. The structural model suggests that the interaction between the negatively charged sulfotyrosine at P2' and the positively charged Arg^302^ of SBT1.8 plays a crucial role in enzyme‐substrate binding. Furthermore, Ser^333^ of SBT1.8 and Asn at position P3', which is not conserved in other CIF peptides, are also suggested to contribute to specificity (Royek et al., 2022).

## PSK

Phytosulfokine (PSK) is a five‐amino acid peptide including two sulfated tyrosine residues and was initially identified as a growth‐promoting factor required for the growth of low‐density plant cell cultures (Matsubayashi & Sakagami, 1996). PSK is recognized by PSKR, a membrane‐localized LRR‐RK, and PSK signaling is involved in plant growth, development, and early immunity (Matsubayashi, 2014; Matsubayashi et al., 2002; Matsubayashi et al., 2006). PSK is produced from the C‐terminal region of an approximately 80‐amino acid precursor protein via tyrosine sulfation and proteolytic processing (Matsubayashi et al., 2006; Yang et al., 1999).

*A. thaliana* subtilase AtSBT1.1 is suggested to be involved in PSK4 processing (Srivastava et al., 2008). This is based on the observation that *AtSBT1.1* expression correlates with conditions for efficient shoot regeneration in *A. thaliana* tissue culture. In an *in vitro* cleavage assay using a fluorescence‐quenching peptide substrate representing the putative subtilase recognition site in the PSK4 precursor, AtSBT1.1 cleaved the PSK4 precursor at Leu^67^‐His^68^ (RRSLVL‐HTD). This cleavage site is three amino acid residues upstream of the N‐terminus of mature PSK4, suggesting that further N‐terminal processing may be required to generate the mature peptide. Alanine‐scanning experiments revealed that substitution of any amino acid in the peptide substrate reduced the reaction rate, and the most sensitive positions were P2–P4 (Val^66^, Leu^65^, Ser^64^, respectively), which are modestly conserved among the PSK precursors. Consistent with these results, AtSBT1.1 was most specific for cleavage of PSK4. It should be noted that these activities of AtSBT1.1 in PSK4 processing are based on *in vitro* cleavage assays using affinity‐purified AtSBT1.1 and short fluorogenic peptide substrates representing the putative subtilase recognition site in the PSK4 precursor. Therefore, the role of AtSBT1.1 in PSK4 processing *in vivo* has not yet been confirmed.

SBT3.8 was identified as a protease that cleaves the C‐terminus of *A. thaliana* PSK, based on transcriptomic analysis under mannitol‐induced osmotic stress conditions. SBT3.8 cleaves the C‐terminus of proPSK1, and this activity is not observed when the aspartic acid at the P1' position (immediately downstream of the C‐terminus of PSK, Asp^82^) is replaced with alanine. Furthermore, the same result was observed at both pH 5.5 and pH 7.0 (Stührwohldt et al., 2021).

To investigate the role of peptide signaling and proteolytic processing of its precursor in stress‐induced flower drop in tomato, transgenic plants overexpressing subtilases were analyzed. This study revealed that plants overexpressing phytaspase 2 (*Sl*Phyt2; *Sl*SBT1.13b) exhibited premature flower abscission. Phytaspases are proteases of the subtilase family that display strict Asp specificity for the cleavage of their substrates. The substrate specificity of *Sl*Phyt2, characterized by Asp selectivity at P1 (immediately upstream of the scissile bond) and a preference for hydrophobic amino acids both upstream and downstream of the cleavage site (P2, P3, and P2'), suggests that PSK precursors are likely substrates for *Sl*Phyt2. A synthetic extended PSK peptide with five precursor‐derived amino acids added to the N‐terminus can be cleaved by *Sl*Phyt2, releasing the mature PSK peptide. This cleavage is aspartic acid‐dependent at P1; substitution of aspartic acid with alanine conferred resistance to cleavage by *Sl*Phyt2 (Reichardt et al., 2020).

## EPF

Epidermal patterning factor 1 (EPF1) and EPF2 are secreted cysteine‐rich peptides that regulate stomatal formation. EPF2, which is expressed in meristemoid mother cells (MMCs) and meristemoids, inhibits neighboring cells from adopting the MMC fate, thereby limiting the density of the stomatal lineage (Hara et al., 2009; Hunt & Gray, 2009). EPF2 is perceived by the LRR‐RK ERECTA, and its co‐receptor TOO MANY MOUTHS (Lee et al., 2012; Nadeau & Sack, 2002; Shpak et al., 2005). EPF/EPF‐like (EPFL) family genes, including EPF2, encode proteins with an N‐terminal signal peptide and a C‐terminal domain containing conserved cysteine residues that are predicted to be proteolytically processed into mature peptides (Kondo et al., 2010; Lee et al., 2012; Ohki et al., 2011; Sugano et al., 2010). It has been demonstrated that SBT5.2, a subtilase abundant in the leaf apoplast, cleaves a fluorescence‐quenching substrate containing the predicted cleavage site of EPF2 *in vitro* (Engineer et al., 2014). The *in vitro* cleavage site is downstream of Asp^75^ (SKNGGVEMEMYPTGSSLPD‐CSYACGACSPC), which is seven amino acid residues downstream of the Stomagen cleavage site (Kondo et al., 2010; Sugano et al., 2010). The cleavage activity of SBT5.2 was not observed with the sequences derived from EPF1 and Stomagen. It remains unconfirmed whether similar cleavage occurs *in vivo* (Engineer et al., 2014).

## RALF

Rapid alkalinization factors (RALFs) are secreted cysteine‐rich peptides with two disulfide bonds. RALF peptides are recognized by receptor kinases of the *Catharanthus roseus* RLK1‐like (CrRLK1L) family and co‐receptor LORELEI (LRE)‐like glycosylphosphatidylinositol (GPI)‐anchored proteins (LLGs) and are involved in various biological processes, including growth, development, and biotic and abiotic stress responses (Blackburn et al., 2020; Haruta et al., 2014; Xiao et al., 2019). RALF was initially identified as a polypeptide that caused the rapid alkalinization of the medium of the tobacco suspension‐cultured cells, and it was revealed that RALFs are ubiquitous in plants (Pearce et al., 2001). Tobacco RALF is derived from the C‐terminal region of a 115‐amino acid precursor, and an Arg‐Arg motif is present just two residues upstream of the N‐terminus of the mature peptide, suggesting that proteases with dibasic substrate specificity may be involved in the proteolytic processing of RALF from its precursor (Pearce et al., 2001). However, subsequent studies demonstrated that this assumption does not apply universally to RALF processing. The *Arabidopsis* AtRALF23 gene encodes a 138‐amino acid precursor protein, with the “RRxL” motif located immediately upstream of the N‐terminus of the mature RALF. This motif was reported to be recognized by the subtilase AtS1P/SBT6.1, an ortholog of the mammalian site‐1 protease (S1P). Analysis of AtS1P mutants demonstrated that AtS1P is required for AtRALF23 processing. *In vitro* peptide cleavage assays revealed that AtS1P cleaves immediately upstream of the N‐terminus of mature RALF23 (INRRIL‐ATRRY). This cleavage activity is lost when RRIL is substituted with GGIL (Srivastava et al., 2009). It should be noted that only 11 of the 35 genes encoding RALF in *A. thaliana* possess S1P cleavage sites (Stegmann et al., 2017).

## SYSTEMIN

Systemin, a polypeptide consisting of 18 amino acids, is the first plant peptide ligand isolated from tomato (*Solanum lycopersicum*) leaves. It is involved in inducing the wound‐induced systemic defense response (Pearce et al., 1991). Systemin is perceived by LRR‐RKs SYSTEMIN RECEPTOR 1 (SYR1) and SYR2, thereby conferring defense against herbivorous insects (Wang et al., 2018). Systemin is a non‐secreted peptide and is generated from a 200‐amino acid precursor protein, prosystemin, via proteolytic processing (McGurl et al., 1992). In this precursor, systemin is flanked by aspartate residues, which are conserved in prosystemins from other Solanaceae species (Constabel et al., 1998; McGurl et al., 1992). Therefore, phytaspase, an Asp‐specific plant subtilase, is a strong candidate for the enzyme responsible for systemin processing. Beloshistov *et al*. isolated and characterized two tomato phytaspases, *Sl*phytaspase‐1 (*Sl*Phyt‐1) and *Sl*phyt‐2, and demonstrated that *Sl*phyt‐1 and ‐2 cleaved prosystemin at the C‐termini of both Asp residues flanking systemin *in vitro*. Since the N‐terminal Asp is two residues upstream of the N‐terminus of mature systemin, the peptide generated by *Sl*phyt is Leu‐systemin, with leucine added at the N‐terminus. Leu‐systemin retains biological activity, though significantly weaker than that of mature systemin, suggesting it may require further N‐terminal processing (Beloshistov et al., 2018). Indeed, the *in vivo* formation of Leu‐systemin has been demonstrated, confirming the involvement of phytaspases in prosystemin processing (Yan et al., 2023). However, the tomato genome contains twelve putative phytaspases, at least five of which, including *Sl*Phyt‐1 and ‐2, have been reported to exhibit a substrate preference for Asp in P1 position (Reichardt et al., 2018). Thus, it remains unclear which of these phytaspases is responsible for processing prosystemin *in vivo*. Furthermore, *in vivo* detection of Leu‐systemin emphasized the need for further trimming of the N‐terminus by a leucine aminopeptidase. Leucine aminopeptidase A (Lap‐A), which cleaves the N‐terminal leucine of Leu‐systemin and is induced by wounding, may be involved in this process (Gu & Walling, 2000).

## PEP

Plant elicitor peptides (Peps) are endogenous elicitor peptides that induce defense responses. Peps are perceived by the membrane‐localized LRR‐RKs PEP RECEPTOR 1 (PEPR1) and PEPR2, and the co‐receptor BRI1‐ASSOCIATED KINASE1 (BAK1), leading to the induction of immune‐like responses (Krol et al., 2010; Tang et al., 2015; Yamaguchi et al., 2006; Yamaguchi et al., 2010). Peps are derived from the C‐terminus of the precursor proteins, PROPEPs. For example, Pep1 is a 23‐amino acid peptide cleaved from the C‐terminus of the 92‐amino acid precursor PROPEP1 (Huffaker et al., 2006). A pharmacological approach using inhibitors revealed that metacaspases are involved in PROPEP1 processing. The cysteine protease METACASPASE 4 (MC4) was identified as the enzyme that cleaves PROPEP1 downstream of the conserved arginine (Arg^69^), releasing PEP1. This cleavage is Arg‐specific. A model has been proposed in which loss of plasma membrane integrity due to damage leads to an increase in intracellular Ca^2+^, which binds to the MC4 zymogen and induces its activation through autocatalytic cleavage, leading to PROPEP1 cleavage. Importantly, because the exogenous PROPEP1 fragment longer than Pep1 displays biological activity similar to that of Pep1, a more important role of MC4‐mediated cleavage is to release Pep1 from PROPEP1 localized on the tonoplast membrane, rather than to obtain a mature peptide of exact size (Hander et al., 2019).

## SCOOP

Serine‐rich endogenous peptides (SCOOPs) are serine‐rich peptides induced by stress (Gully et al., 2019). SCOOPs are perceived by the LRR‐RK MALE DISCOVERER 1‐INTERACTING RECEPTOR‐LIKE KINASE 2 (MIK2) and the co‐receptor BAK1, leading to the induction of immune responses (Hou et al., 2021; Rhodes et al., 2021). SCOOP peptides are derived from the conserved C‐terminal motif of the precursor PROSCOOP, which possesses an N‐terminal signal peptide (Gully et al., 2019; Hou et al., 2021). Yang *et al*. revealed that the *A. thaliana* genome contains at least 50 *PROSCOOP* genes. Many of these PROSCOOPs possess the “RxLx/RxxL” motif, known as a recognition site for some SBTs including SBT6.1 and SBT3.5. Based on transcriptional co‐upregulation with *MIK2* under various conditions, substrate specificity, and PROSCOOP12 cleavage assays, SBT3.5 was identified as the protease that cleaves PROSCOOP12. On the contrary, some PROSCOOPs lack the “RxLx/RxxL” motif and instead contain a “VWD” motif. PROSCOOP20, belonging to such a subgroup, is cleaved by the aspartate‐dependent subtilase SBT3.8 and its homologs SBT3.6 and SBT3.9 (Yang et al., 2023).

## flg22

The 22‐amino acid peptide flg22, derived from the bacterial flagellar protein flagellin, is the most extensively studied MAMP (Felix et al., 1999). In *A. thaliana*, the LRR‐RK FLAGELLIN SENSING 2 (FLS2) directly recognizes flg22, leading to the initiation of immune signaling (Chinchilla et al., 2006; Gómez‐Gómez & Boller, 2000; Sun et al., 2013; Zipfel et al., 2004). Peptide MAMPs, such as flg22, are predicted to be released from microbial precursor proteins by host plant proteases (Chen et al., 2024).

Recently, a biochemical analysis combining proteomics, a fluorescence‐quenching peptide substrate, and native two‐dimensional polyacrylamide gel electrophoresis identified two *A. thaliana* subtilases, SBT5.2 and SBT1.7, as the specific proteases responsible for C‐terminal cleavage of flg22 (Matsui et al., 2024). Recombinant SBT5.2 and SBT1.7 cleave at the C‐terminus of the flg22 domain of flagellin. This activity was confirmed not only for the amino acid sequence of flagellin from *Pseudomonas syringae* pv. *tomato* DC3000 (GLQIA‐TKITS), but also for those from *Pseudomonas aeruginosa* (GLQIA‐NRLTS) and *Xanthomonas axonopodis* pv. *citri* (GLAIS‐ERFTT). These results indicate broad substrate specificity of these proteases, which may ensure that FLS2 recognizes a wide range of pathogens. Similarly, it has been reported that SBT5.2 in *Nicotiana benthamiana* cleaves flagellin from *Pseudomonas syringae* pv. *tabaci* 6605 at the ends of flg22 and releases flg22 from flagellin (Buscaill et al., 2024). Alanine‐scanning experiments revealed that isoleucine at the P2 position (Ile^50^) is critical for efficient cleavage by SBT5.2 (Matsui et al., 2024). Interestingly, SBT5.2 and SBT1.7 cleave not only the C‐terminus of flg22 (Ala^51^‐Thr^52^) but also within the flg22 domain (Asn^39^‐Ser^40^), which impairs the activity of flg22. This dual activity of SBT5.2 and SBT1.7 may reflect an evolutionary “hide‐and‐seek” between plants and bacteria. In this way, the identification of molecules and analysis of their cleavage activity can reveal unexpected biological significance (Matsui et al., 2024). The roles of SBT5.2 in degradation of flg22 have also been demonstrated in a study using *Nicotiana benthamiana* and *Pseudomonas syringae* pv. *tabaci* 6605 (Buscaill et al., 2024).

## GENERAL INSIGHTS INTO SUBTILASES AS KEY INITIATORS OF PEPTIDE LIGAND‐RECEPTOR SIGNALING

As described in the sections above, most proteases identified as being involved in the release of peptide ligands belong to the subtilase family, a group of subtilisin‐like serine protease. Subtilases have been identified as proprotein convertases responsible for the processing of proproteins and prohormones with a clear preference for basic residues in mammals and yeast (Seidah & Chrétien, 1999). Additionally, subtilases form large gene families with 56 members in *A. thaliana* (Rautengarten et al., 2005), 56 in *N. benthamiana* (Grosse‐Holz et al., 2018), and 82 in tomato (Reichardt et al., 2018). Thus, subtilases have been actively studied as promising candidates for peptide processing enzymes in plants. Furthermore, the development of class‐specific inhibitors has advanced research (Schardon et al., 2016; Stührwohldt, Ehinger, et al., 2020; Stührwohldt, Scholl, et al., 2020). In this section, we integrate and analyze the information on subtilases presented above to discuss their activation, substrate specificity, and coordinated functions in peptide ligand release.

### Regulation of proteolytic processing

Proteolytic processing of peptide ligands serves as the initiation point of signaling; therefore, the activity of the proteases responsible for this process can be subject to strict regulation. Some proteases are regulated at the transcriptional level. For example, SBT5.2, which functions in the regulation of stomatal density in response to CO_2_ concentration, is transcriptionally upregulated along with its substrate EPF2 under elevated CO_2_ conditions (Engineer et al., 2014). SBT3.5, which cleaves PROSCOOP, is transcriptionally induced under stress conditions (Yang et al., 2023). SBT3.8 in *A. thaliana* and *Sl*Phyt in tomato are also induced along with PSK under drought stress, consistent with their roles in PSK maturation (Reichardt et al., 2020; Stührwohldt et al., 2021). These findings suggest that initiation of peptide signaling in response to stress may require induction of the corresponding proteases.

However, transcriptional induction is rather an exception as a regulatory mode for subtilases. Subtilases are produced as zymogens (inactive proenzymes), which are often activated autocatalytically through cleavage of their inhibitory prodomains. Therefore, protease activities are usually regulated through post‐translational factors.

**Figure 3:**
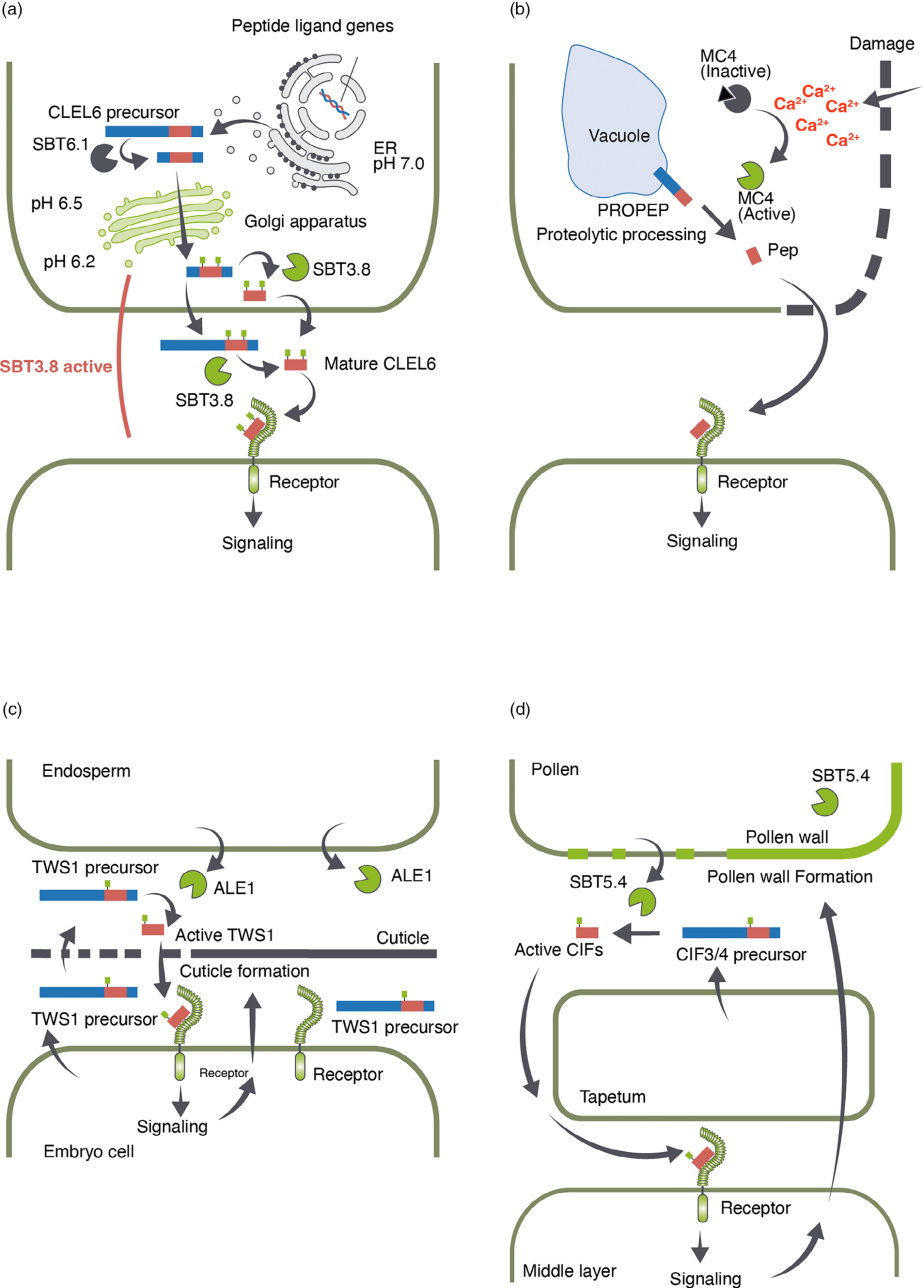
Regulation of protease activity in peptide ligand generation. (a) pH‐dependent regulation of CLEL6 processing by SBT3.8. Since SBT3.8 cleaves the CLEL6 precursor under acidic conditions, cleavage is suggested to occur in the post‐Golgi compartment or the apoplast (Stührwohldt, Scholl, et al., 2020). (b) Damage‐induced Ca^2+^ influx‐dependent release of Pep. Ca^2+^ influx through the damaged regions of the cell (indicated by dashed lines) activates MC4, which cleaves PROPEP to release Pep. The released, mobile Pep exits the cell through the damaged region into the extracellular space, binds to receptors on neighboring cells, and initiates signaling (Hander et al., 2019). (c) Monitoring the integrity of the embryonic cuticle through the spatial segregation of proteases and substrates. The left side shows the situation before the cuticle gap is completely filled. ALE1 and TWS1 diffuse and meet at the interface between endosperm and embryonic cells through the gap, where TWS1 processing occurs. The right side shows the situation with the cuticle intact. Proteases and substrates are segregated, preventing TWS1 maturation and attenuating signaling (Doll et al., 2020). (d) Regulation of pollen wall formation by protease segregation. The left side shows incomplete pollen wall. Pollen‐derived SBT5.4 processes tapetum‐derived CIF precursors to generate mature CIF3/4, thereby promoting pollen wall formation. A complete pollen wall, as seen on the right, inhibits interaction between SBT5.4 and its substrates, attenuating the signal (Truskina et al., 2022).

One such factor is the ionic environment. For example, cleavage of CLEL6 by SBT3.8 occurs at acidic pH rather than at neutral pH, suggesting that processing takes place only during the later stages of the secretory pathway (Figure 3a) (Stührwohldt, Scholl, et al., 2020). By contrast, cleavage of PSK1 by SBT3.8 appears to occur regardless of pH (Stührwohldt et al., 2021), indicating that the pH dependence of a single enzyme can vary depending on the substrates. These observations suggest that, in the case of SBT3.8, pH may influence substrate selectivity rather than the removal of prodomain. As will be discussed later, some proteases have a broad range of substrate specificities, and the relationship between proteases and substrates is not one‐to‐one. Variations in pH sensitivity depending on the substrate could contribute to the selective activation and switching of different signaling pathways. In addition, although not a subtilase, MC4 is activated in a Ca^2+^‐dependent manner to release Pep1 (Hander et al., 2019). This results in specific production of Pep during tissue damage, when there is a prolonged influx of Ca^2+^ (Figure 3b). Changes in the ionic environment often accompany injury or immune responses. Therefore, the regulation of proteases by ionic environment can serve as a trigger for peptide ligand‐receptor signaling in response to these environmental stimuli.

The second mechanism involves post‐translational modification of the substrate. The inhibitory cleavage within the CLE40 peptide by SBT4.13 is prevented by proline hydroxylation upstream of the cleavage site (Stührwohldt, Ehinger, et al., 2020). By contrast, the N‐terminal cleavage of TWS1 by SBT1.8 requires tyrosine sulfation downstream of the cleavage site (Royek et al., 2022). While post‐translational modifications of peptide hormones are sometimes necessary for receptor interactions, they may also play a crucial role in ensuring proper control of processing. The spatiotemporal relationships between post‐translational modification enzymes and processing proteases, such as the order of their actions and their localization (e.g., intracellular compartments or the extracellular space), and how these regulatory mechanisms are influenced by developmental and environmental factors remain largely unknown and require further investigation. Identification of such a novel mode of regulation provides an important foundation for future studies of various biological processes mediated by peptide ligands and their receptors in plants.

Lastly, spatial regulation of peptide‐protease interactions represents another regulatory mechanism. CIF3, CIF4, and TWS1 are expressed in cell types distinct from their corresponding processing proteases. The substrate and enzyme are secreted and meet in the apoplast to generate mature CIF peptides. The CIF signaling pathway forms a barrier between precursor‐producing cells and protease‐producing cells. Once the barrier is complete, the substrate and enzyme no longer meet, thereby terminating the signal (Figure 3c,d) (Doll et al., 2020; Truskina et al., 2022). This highlights that, in addition to peptide ligands and receptors, processing proteases play an important role as a third key component in determining tissue boundaries.

### Substrate specificity

Identification of proteases provides valuable insights into their substrate specificities. Mammalian subtilases involved in the processing of peptide precursors often cleave at single and/or paired basic residues (Seidah & Chrétien, 1999). By contrast, no typical consensus cleavage motif has been identified for plant subtilases; instead, cleavage occurs at diverse sites, as summarized in Table 1. Phytaspases, such as SBT3.8 and *Sl*Phyt, exhibit strong specificity for aspartate residues at the cleavage site (Beloshistov et al., 2018; Reichardt et al., 2020; Stührwohldt et al., 2021; Stührwohldt, Scholl, et al., 2020). Some subtilases, including AtS1P/SBT6.1 and SBT3.5, cleave at the “RxxL/RxLx” motif, which corresponds to the canonical consensus motif cleaved by the mammalian site‐1 protease (S1P) (Ghorbani et al., 2016; Yang et al., 2023). Although some subtilases share similar substrate preferences, these motifs do not represent a general consensus. This diversity indicates that plant subtilases can target a wide range of sequences and are potentially involved in the processing of various peptide ligands.

**Table 1:** The substrates and cleavage sites of proteases discussed in this review

Species	Protease	Substrate	Cleavage site		References
*A. thaliana*	SBT4.12	IDA	FG**Y**L**P**K↓GVPIPP	Lys55‐Gly56	Schardon et al. (2016)
		IDA	PSKRHN↓SFVNSL	Asn69‐Ser70	Schardon et al. (2016)
	SBT4.13	IDA	FG**Y**L**P**K↓GVPIPP	Lys55‐Gly56	Schardon et al. (2016)
		IDA	PSKRHN↓SFVNSL	Asn69‐Ser70	Schardon et al. (2016)
		CLE40	RQV**PT** ↓G↓SDPLHH↓K↓HIPFTP	Thr66‐Gly67, Gly67‐Ser68, His73‐Lys74, Lys74‐His75	Stührwohldt, Ehinger, et al. (2020)
	SBT5.2	IDA	FG**Y**L**P**K↓GVPIPP	Lys55‐Gly56	Schardon et al. (2016)
		IDA	PSKRHN↓SFVNSL	Asn69‐Ser70	Schardon et al. (2016)
		EPF2	**T**GS**SL**P**D**↓C**S**Y**AC**G	Asp75‐Cys76	Engineer et al. (2014)
		Flagellin DC3000	GLQ**I**A↓TKITS	Ala51‐Thr52	Matsui et al. (2024)
		Flagellin aeruginosa	GLQIA↓NRLTS	Ala51‐Asn52	Matsui et al. (2024)
		Flagellin Xac	GLAIS↓ERFTT	Ser51‐Glu52	Matsui et al. (2024)
		Flagellin DC3000	SGLKIN↓SAKDDA	Asn39‐Ser40	Matsui et al. (2024)
	SBT1.4	CLE40	RQV**PT** ↓G↓SDPLHH	Thr56‐Gly57, Gly57‐Ser58	Stührwohldt, Ehinger, et al. (2020)
	SBT1.7	CLE40	RQV**PT** ↓G↓SDPLHH↓KHIPFTP	Thr66‐Gly67, Gly67‐Ser68, His73‐Lys74	Stührwohldt, Ehinger, et al. (2020)
		Flagellin DC3000	GLQ**I**A↓TKITS	Ala51‐Thr52	Matsui et al. (2024)
		Flagellin DC3000	SGLKIN↓SAKDDA	Asn39‐Ser40	Matsui et al. (2024)
	SBT6.1	RGF6/GLV1/CLEL6	AA**RRLR**↓SHKHHH	Arg34‐Ser35	Ghorbani et al. (2016)
	SBT6.1	RGF6/GLV1/CLEL6	ER**RRA**↓**L**↓GGVETG	Ala57‐Leu58, Leu58‐Gly59	Ghorbani et al. (2016)
		RALF23	IN**RR**IL↓ATRRY	Leu88‐Ala89	Srivastava et al. (2009)
	SBT3.5	PROSCOOP12	TG**RRLM**↓GSGASG	Met45‐Gly46	Yang et al. (2023)
	SBT3.8	PROSCOOP20	KE**VWD**Q↓TLLR**↓**DLKIGA	Gln45‐Thr46, Arg49‐Asp50	Yang et al. (2023)
		PSK1	DYIYTQ↓ **D**LNLSP	Gln81‐Asp82	Stührwohldt et al. (2021)
		RGF6/GLV1/CLEL6	EEVVVM↓ **D**YPQPH	Met70‐Asp71	Stührwohldt, Scholl, et al. (2020)
	SBT5.4	CIF4	PGPVPH↓GSL	His89‐Gly90	Truskina et al. (2022)
	SBT2.4 (ALE1)	TWS1	PGPIE**H** ↓**G**TPLNP	His54‐Gly55	Doll et al. (2020)
	SBT1.8	TWS1	PGPIEH↓GTPLNP	His54‐Gly55	Royek et al. (2022)
		TWS1	MKVGLE↓D**Y**NFPV	Glu31‐Asp32	Royek et al. (2022)
	SBT1.1	PSK4	RR**SLV**L↓HTD	Leu67‐His68	Srivastava et al. (2008)
	MC4	PROPEP	VVVTS**R**↓ATKVK	Arg69‐Ala70	Hander et al. (2019)
Tomato	SlPhyt1	Systemin	IIVRE**D**↓LAVQSK	Asp177‐Leu178	Beloshistov et al. (2018)
			PKMQT**D** ↓NNKL	Asp196‐Asn197	Beloshistov et al. (2018)
	SlPhyt2	Systemin	IIVRE**D**↓LAVQSK	Asp177‐Leu178	Beloshistov et al. (2018)
			PKMQT**D** ↓NNKL	Asp196‐Asn197	Beloshistov et al. (2018)
		SlPSK	EAFL**D**↓YIYTQ	Asp80‐Tyr81 (SlPSK1)	Reichardt et al. (2020)
*N. benthamiana*	SBT5.2	Flagellin Pta6605	STSM↓TRLS	Met29‐Thr30	Buscaill et al. (2024)
		Flagellin Pta6605	LKIN↓SAKD	Asn39‐Ser40	Buscaill et al. (2024)
		Flagellin Pta6605	LQIA↓TKITS	Ala51‐Thr52	Buscaill et al. (2024)

For each protease‐substrate pair, the reported cleavage site sequences are shown. The symbol ↓ indicates the scissile bond. Amino acids shown in bold represent those subjected to substitution experiments, and underlined residues denote the mature peptide ligand site. Single‐letter abbreviations for the amino acid residues are as follows: A, Ala; C, Cys; D, Asp; E, Glu; F, Phe; G, Gly; H, His; I, Ile; K, Lys; L, Leu; M, Met; N, Asn; P, Pro; Q, Gln; R, Arg; S, Ser; T, Thr; V, Val; W, Trp; and Y, Tyr. The right column indicates the positions of the amino acid residues flanking the scissile bond, based on their numbering in the precursor protein.

Notably, although plant subtilases do not have a clear consensus cleavage motif, a possible common feature is that substrate recognition tends to depend not on a single amino acid, but on an extended sequence motif flanking the cleavage site. In addition, as mentioned above, post‐translational modifications can influence protease recognition and cleavage specificity. Substrate preferences that depend on multiple amino acids confer high substrate selectivity, which is essential for processing proteases that act as key initiators of peptide ligand‐receptor signaling and therefore require tight regulation.

### Coordinated function to release peptide ligands

As shown in Table 1, substrates and enzymes do not necessarily have a one‐to‐one relationship; a single protease can cleave multiple substrates, and conversely, multiple proteases can function redundantly on a single substrate.

In the processing of IDA, CLE40, and SCOOP20, it has been shown that multiple subtilases can cleave the same cleavage site (Schardon et al., 2016; Stührwohldt, Ehinger, et al., 2020; Yang et al., 2023). Other peptides are likely to be processed by multiple subtilases, although this has not yet been confirmed, suggesting that such redundancy is common in peptide ligand processing. This redundancy may reflect the importance of releasing peptide ligands from their precursors. However, this also complicates the observation of phenotypes in single subtilase mutants, posing an obstacle to elucidating protease functions.

For the maturation of some peptides, consecutive processing steps are required. CLEL6 undergoes pre‐processing by SBT6.1 in the early Golgi compartment, followed by N‐terminal cleavage and final activation by SBT3.8 in the acidic environment of the post‐Golgi compartments, including the apoplast (Figure 3a) (Stührwohldt, Scholl, et al., 2020). Pre‐processing by SBT6.1 appears to be required for passage through the secretory pathway, and processing by SBT3.8 occurs only under acidic conditions. This two‐step processing mechanism may act as a checkpoint, ensuring that only correctly processed precursors are ultimately activated by SBT3.8 and other aspartate‐dependent subtilases. Another example is the maturation of systemin. Prosystemin is cleaved by phytaspases, which are aspartate‐dependent subtilases, at one residue upstream of mature systemin, generating systemin with an additional leucine at its N‐terminus (Leu‐systemin) (Beloshistov et al., 2018; Yan et al., 2023). Leu‐systemin displays bioactivity, but its activity is weaker than that of mature systemin (Beloshistov et al., 2018). For the generation of fully active peptides, trimming of the N‐terminal leucine by aminopeptidase(s) is required. The stepwise processing that generates two peptides with different activity levels may represent a mechanism for modulating the intensity of systemin signaling.

Interestingly, a single enzyme can cleave a substrate at two distinct sites. For example, subtilases cleave the precursors of CLE40 and flg22, releasing the mature peptide ligands from their precursors while inactivating them through internal cleavage (Buscaill et al., 2024; Matsui et al., 2024; Stührwohldt, Ehinger, et al., 2020). Internal cleavage of CLE40 is inhibited by proline hydroxylation in vicinity of the scissile bond (Stührwohldt, Ehinger, et al., 2020). Similarly, flg22 is suggested to be protected from internal cleavage through binding to its receptor FLS2 (Buscaill et al., 2024). These findings suggest that subtilases not only initiate peptide ligand‐receptor signaling but also regulate peptide ligand levels by degrading them depending on availability as ligands.

## CONCLUDING REMARKS AND FUTURE PERSPECTIVES

In recent years, an increasing number of proteases have been identified as being involved in the release of peptide ligands from their precursors in plants. To date, the majority of identified peptide ligands have been shown to be released by subtilases, and this review has described representative examples of them. Finally, we discussed the features of subtilases that are critical for their role as key initiators of peptide ligand‐receptor signaling, including their diverse regulatory mechanisms, broad yet highly selective substrate specificity, and cooperative modes of action. It should be noted that while these characteristics may be necessary for subtilases to function as initiators, they are not unique to subtilases. Indeed, other proteases have also been shown to participate in the processing of peptide ligands. Some cysteine proteases have been shown to cleave certain peptides from their precursors, classified as damage‐associated molecular patterns (DAMPs)—molecules released upon cellular damage that trigger immune responses—such as PEP, and although not detailed here, GRIM REAPER (GRI), *Zea mays* immune signaling peptide 1 (Zip1), and cysteine‐rich secretory protein, antigen 5, and pathogenesis‐related 1 protein (CAP)‐derived peptide 9 (CAPE9) (Chen et al., 2023; Wrzaczek et al., 2015; Ziemann et al., 2018). Moreover, exoprotease activities such as those of SOL1 and Lap‐A are also involved in peptide processing (Gu & Walling, 2000; Tamaki et al., 2013). Aspartic proteases EGG CELL‐SPECIFIC1 (ECS1) and ECS2 cleave the cysteine‐rich peptide LURE1, a pollen tube attractor, thereby preventing the simultaneous penetration of ovules by multiple pollen tubes (Yu et al., 2021). Although this cleavage does not involve the release of peptide ligands, it represents an example of aspartic proteases involved in the cleavage of peptide ligands. In addition, ECS1 and ECS2 play an important role in promoting preferential fertilization of egg cells (Jiang et al., 2022). However, the substrate involved in this process has not yet been identified. It is likely that the substrates of ECS1 and ECS2 are secreted from the egg cells (Jiang et al., 2022), suggesting that ECS1 and ECS2 may participate in the processing of egg cell–derived secreted peptides.

As previously noted, peptide ligand‐mediated signaling has been increasingly recognized as a widespread phenomenon in land plants. Synthetic peptide treatment assays in the liverwort *Marchantia polymorpha* suggest that the bioactive structures of CLE peptides are similar between *M. polymorpha* and *A. thaliana* (Hirakawa et al., 2020). However, little is known about the *in vivo* processing of peptide ligands in non‐flowering plants. If peptide ligands are conserved, are the corresponding processing proteases also conserved? With the increasing availability of genomic information, it is now possible to elucidate the characteristics of processing proteases in land plants.

In this way, processing proteases may be hidden not only within the subtilase family in vascular plants but also across other protease groups in land plants. To fully understand the repertoire of proteases used by plants for peptide ligand processing, it is desirable to identify processing proteases that have not yet been characterized.

The identification of processing proteases has often relied on analyses of mutants related to specific biological phenomena or on expression pattern analyses. However, plants possess hundreds of protease genes (Rawlings et al., 2018), many of which can function redundantly. Additionally, many proteases are regulated not only at the level of gene expression but also, as mentioned earlier, through mechanisms that control their activity. These factors limit the effectiveness of conventional methods such as mutant analysis and expression profiling. In recent years, biochemical approaches—such as loss‐of‐function methods using SBT‐specific inhibitors (Schardon et al., 2016) and activity‐based screening methods (Matsui et al., 2024)—have led to the identification of new proteases. With the development of such novel methods, the identification of peptide ligand‐processing proteases is expected to advance further, which will ultimately facilitate a deeper understanding of peptide ligand‐receptor signaling mechanisms.

## AUTHOR CONTRIBUTIONS

SM and YH conducted the conceptualization of this review and wrote the manuscript. SM created the figures and table.

## CONFLICT OF INTEREST STATEMENT

The authors declare no competing interests.

## Data Availability

Data sharing not applicable to this article as no datasets were generated or analysed during the current study.
